# Cell-autonomous action of *Slit2* in radial migration of cortical projection neurons

**DOI:** 10.3389/fnmol.2024.1505434

**Published:** 2024-12-02

**Authors:** Tian Jiang, Guozhen Niu, Chunping Wu, Xiaomeng Tu, Jian Xiao, Xue Li, Jie-Guang Chen, Huateng Cao

**Affiliations:** ^1^Department of Clinical Laboratory, The Affiliated Wenling Hospital (The First People’s Hospital of Wenling), Wenzhou Medical University, Wenling, China; ^2^State Key Laboratory of Optometry, Ophthalmology and Vision Science, School of Ophthalmology and Optometry and Eye Hospital, Wenzhou Medical University, Wenzhou, China; ^3^Zhejiang Provincial Key Laboratory of Optometry and Ophthalmology, School of Ophthalmology and Optometry and Eye Hospital, Wenzhou Medical University, Wenzhou, China; ^4^Department of Ophthalmology, Tongji Hospital, Tongji University School of Medicine, Shanghai, China; ^5^Nanchang People's Hospital, Nanchang, China

**Keywords:** SLIT2, ROBO2, radial migration, cortical development, morphogenesis, neuronal polarization, autocrine

## Abstract

Neuronal radial migration is a fundamental process for cortical development, the disruption of which causes neurological and psychiatric dysfunctions. SLIT2 plays diverse functions in brain development and is a well-known axon guidance molecule. In this study, we investigated the radial migration of projection neurons in the developing cerebral cortex by *in utero* knockdown (KD) of *Slit2* in mice. KD of *Slit2* did not interfere with the neurogenesis and fate-determination but led to the accumulation of the transfected cells in the intermediate zone (IZ), suggesting that the expression of *Slit2* is crucial for the radial migration of the cortical neurons. KD of *Slit2* hindered the transition of cells from a multipolar to a bipolar shape, which is necessary for glia-guided locomotion. Interestingly, reducing Slit2 did not affect the migration of neighboring untransfected cells, indicating a cell-autonomous action by SLIT2. In addition, the action of SLIT2 KD was mimicked by a dominant negative mutant of ROBO2, a canonical membrane receptor of SLIT2, supporting that SLIT2 acted locally as a secretory molecule. Our results suggest that SLIT2 is indispensable for the radial migration of cortical neurons through an autocrine signaling mechanism.

## Introduction

The cerebral cortex is a highly ordered brain structure with neurons organized into six distinct layers. The projection neurons in each layer arise from neural progenitors residing in the ventricular zone (VZ) and subventricular zone (SVZ) during the embryonic period, and arrive in corresponding layers in an “inside-out” fashion through sophisticated radial migration (Angevine and Sidman, 1961; Rakic, 1974). The newborn multipolar neurons in the VZ move toward the SVZ and the lower intermediate zone (IZ), where they make small tangential displacements and undergo a re-polarization process by extending a leading and a trailing process. The bipolar-shaped neurons attach to radial glial cells and migrate through the upper IZ toward their destination in the cortical plate (CP) by glia-guided locomotion (Nadarajah and Parnavelas, 2002; Tabata and Nakajima, 2003; Noctor et al., 2004). Neuronal migration is a fundamental process of cortical formation, and disturbance of any steps of this process can cause the misplacement of neurons and result in disorganized cortical lamination and circuit formation. Disruption of neuronal migration is the underlying pathophysiology of multiple neurological and psychiatric dysfunctions (Clark, 2002; Bielas et al., 2004; Gressens, 2005; Guerrini and Filippi, 2005; McManus and Golden, 2005).

A key step affecting radial migration is the transition from multipolar to bipolar morphology. This polarization process depends on remodeling intracellular cytoskeleton and microtubule dynamics, which are regulated by extracellular cues and intracellular signaling molecules. For example, PI3-Kinase-activated signaling networks (PI3K/Akt/GSK-3β/CRMP-2 and PI3K/Cdc42/Par complex/Rac1) have been identified as critical intracellular events controlling the polarization of cortical neurons (Arimura and Kaibuchi, 2005; Solecki et al., 2006; Yoshimura et al., 2006; Witte and Bradke, 2008). Many secretory extracellular molecules (e.g., netrins, semaphorins, reelin, TGF-ß, and neurotrophins) have also been shown to regulate neuronal radial migration (Sakakibara and Hatanaka, 2015; Takano et al., 2015).

SLIT2 is a secretory glycoprotein that binds to its canonical receptor Roundabout (Robo) and activates the intracellular signaling cascade, leading to the alteration of actin polymerization and microtubule cytoskeleton (Chedotal, 2007). Studies in rodents found that Slit2 is an axon guidance molecule (Seeger et al., 1993; Brose et al., 1999; Farmer et al., 2008), and transgenic mice with Slit2 overexpression exhibit depression and anxiety-like behavior (Huang et al., 2020). In humans, SLIT2 has been identified as a candidate gene for developmental dyslexia (Poelmans et al., 2011), indicating its involvement in neural development disorders (Sherchan et al., 2020). The downstream molecules of the SLIT2-Robo signaling pathway (e.g., GTPase activating proteins, Rho family of small GTPases, Cdc42, and intracellular calcium), were reported modulating the radial migration of cortical neurons in rodents (Ypsilanti et al., 2010; Jossin and Cooper, 2011). However, whether SLIT2 acts diffusively or locally in the central nervous system is unclear.

In this study, we inhibited *Slit2* expression in the developing cerebral cortex by introducing shRNA via *in utero* electroporation (IUE). Our results indicate that suppression of *Slit2* dramatically impairs radial migration and neuronal re-polarization associated with Golgi reorienting. Interestingly, we found that the inhibitory effect of *Slit2* KD on radial migration did not apply to the nearby control neurons. Furthermore, a truncated ROBO2 induced a dominant negative effect similar to *Slit2* knockdown. These results uncovered that SLIT2 modulates neural radial migration by an autocrine action in the developing neocortex.

## Materials and methods

### Animals

All animal experiments in this study were performed according to the guidelines approved by the Animal Care and Ethics Committee of Wenzhou Medical University and conformed to international guidelines on the ethical use of animals. ICR mice were obtained from Shanghai Laboratory Animal Center of the Chinese Academy of Sciences (Shanghai, China). Embryonic day was numbered starting from E 0.5, the morning when vaginal plug was detected. The day of birth was counted as postnatal day 0 (P0).

### Single cell-RNA sequencing data processing

Published scRNA-seq datasets from GEO (Di Bella et al., 2021; Yim et al., 2024) were collected and re-analyzed using Seurat v4.0.4. Cells met the following criteria were filtered out for downstream processing: (1) detected gene number: >200 and < 9,000; (2) total unique molecular identifier (UMI) counts <60,000; and (3) the percentage of mitochondrial genes <12%. Qualified cells were applied to further analysis. “LogNormalize” (scale factor: 10,000) was used to normalize gene expression in each cell. The proportion of mitochondrial UMIs was regressed out (ScaleData function). The top 2000 variable genes were identified (FindVariableGenes function) and used as input for dimensionality reduction via principal component analysis (PCA) after removing interfering genes including sex-specific genes, immediate early genes, virus-induced genes and 1,000 noise-sensitive genes^59^. “RunPCA” was applied to find the PCs and 25 PCs were used as input for clustering analysis (FindClusters function, resolution = 3). Cell clusters were identified, and each cluster was classified to canonical cell types in brain according to the following markers: Slc32a1 indicates interneurons, Fezf2 and Bcl11b indicate deep-layer projection neurons, Pou3f2 indicates upper-layer fate neurons, Pax6 indicates precursor cells, Eomes indicates intermediate progenitors, Neurod1 indicates mitotic and early-postmitotic neurons and Satb2 indicates postmitotic neurons. FeaturePlot function were used to plot and visualize the expression of target genes in the cell clusters.

### DNA constructs

*Slit2* was amplified by PCR from P0 mouse dorsal telencephalon cDNA library with a forward primer TCAGCCGCTCGAGAGTTGCCCACGGATCTTCTG, and a reverse primer AAGGAAAAAAGCGGCCGCGTCAAGAAGTGTACAACCTTCGC, which at the 5′ end contains XhoI and NotI endonuclease sites, respectively. The PCR product contains full-length coding sequence of Slit2 but lacks the 3′ untranslated region (3’UTR). A truncated ROBO2 without intracellular domain was also created by PCR with a forward GGTGTAGCCAATTGGCCATGAATCCTCTGATGTTTACACTATTATTGCTC and a reversal primer GGTGTAGCGCGGCCGCTCAGTTATCTCTCTTCTTTCTTCTCCAGTACAACCAGATG. All PCR products were cloned into pCAGEN (Addgene), which harbors a CAG promoter for efficient expression *in vivo*.

To suppress the expression of Slit2, small interference RNA (siRNA) were cloned into pSUPER vector under the control of H1 promoter with a loop “TTCAAGAGA” as described previous (Brummelkamp et al., 2002). The following siRNA sequences targeting the Slit2 were obtained from a Sigma database.^1^ The control a scramble sequence does not match with any sequence in the mouse genome by blast search.

Slit2-shRNA-1: GCTACCAGTTTCCAGTATTAA;

Slit2-shRNA-2: GCCTGTCAAACAACTAAGAAA;

Slit2-shRNA-3: GCCTTGTCACACTTAGCGATT;

Slit2-shRNA-4: GCTGGAAAGACTGCGTTTAAA;

Control-shRNA: GAATTTCGTTCCTCGTTTAGAT.

### Cell line culture and transfection

N2a (Neuro-2a) cells were cultured in DMEM/F-12 medium (Thermo Fisher Scientific, Cat# 11320033) supplemented with 10% Fetal Bovine Serum (Thermo Fisher Scientific, Cat# C838T52), and passaged every 3 days according to standard protocols. N2a cells are male cells. The efficacy of shRNA plasmids was tested in N2a cells. Transfection was carried out 24 h after cell plating using Lipofectamine 3,000 (Thermo Fisher Scientific, Cat# L3000015, Invitrogen, United States) according to the manufacturer’s protocols. Total RNA was extracted from N2a cell two days after the transfection using RNeasy Mini Kit (Qiagen). Quantitative PCR was performed using cDNA synthesized from the RNA, and the expression of Slit2 was detected by SYBR fluorescence.

### Western blots

Protein samples of N2a cells were harvested in RIPA lysis buffer (Beyotime, China, Cat# P0013B) 48 h after transfection. Cortical samples were dissected and homogenized in RIPA lysis buffer. Western blot was performed according to standard protocols as previously described. The primary antibodies and dilutions were: rabbit anti-SLIT2 antibody (Abcam Cat# ab7665, RRID:AB_449621, 1:800) and mouse anti-*β*-actin antibody (Cell Signaling Technology Cat# 4967, RRID:AB_330288, 1:1000). Blots were visualized using ECL chemiluminescence substrate (Thermo Fisher Scientific, Cat# 32106) and Amersham Imager 600 (GE Healthcare, Chicago, IL, United States). For example images, brightness/contrast adjustments within linear ranges were made using Fiji/ImageJ across the entire image.

### Immunohistochemistry

To detect gene expression by immunofluorescence (IF), the diseected brains were fixed in 4% PFA, cryoprotected by immersion in 30% sucrose in PBS at 4°C, embedded in OCT compound, and sectioned at 16 μm with a cryostat microtome (Leica). Antigen retrieval was performed to improve the IF detection, if necessary, by heating samples at 90°C in sodium citrate buffer (0.01 M, pH: 6.0) for 5 ~ 10 min. The sections were blocked for 2 h at room temperature in a blocking solution (phosphate-buffered saline, PBS, with 5% Donkey Serum, 1% BSA and 0.3% Triton), then incubated with primary antibodies (diluted in the blocking solution) at 4°Cfor 48 h. The antibodies used in this study include: Anti-GFP (Novus Cat# NB600-308, RRID:AB_10003058, 1:1000), Anti-phospho-Histone H3 (Abcam, 1:300), Anti-Tbr2 (Abcam Cat# ab23345, RRID:AB_778267, 1:400), Anti-Satb2 (Santa Cruz Biotechnology Cat# sc-81376, RRID:AB_1129287, 1:100), Anti-GM130 (BD Biosciences Cat# 610823, RRID:AB_398142, 1:100). Following primary antibody incubation, the cryosections were washed and incubated with Dylight488/549-conjugated secondary antibodies (donkey anti-mouse/rabbit/goat, 1:500, Jackson ImmunoResearch Laboratories) for 2 h at room temperature. Finally, the sections were washed, air-dried, and mounted with *SlowFade^®^* Gold Antifade Mountant with DAPI (Life Technologies) for nuclear labeling. The z-series stacks images of immunostained specimens were scanned by using a Zeiss LSM710 confocal microscope with a Zeiss 0.8 NA 20× lens/40× oil immersion lens at ×0.9 zoom, 1 μm step size, 1,024 × 1,024 resolution, and a scan speed of 600 Hz.

### *In utero* electroporation

*In utero* electroporation (IUE) was performed as previously described (Cao et al., 2020; Jiang et al., 2023). Briefly, pregnant ICR mice were anesthetized by intraperitoneal injection of ketamine (100 mg/kg) and xylazine (10 mg/kg) diluted in sterile 0.9% saline. A laparotomy of 3 cm incision was performed in the low middle abdomen, and the uterus was carefully taken out. Approximately 2 μg of plasmid DNA in Tris-EDTA buffer mixed with 0.025% of FastGreen was delivered into the lateral ventricles with a glass micropipette. Electric pulses (40 mV for 50 ms) were applied to the brains five times at intervals of 950 ms with an electroporator (BTX, T830). Upon completion of injection and electroporation, the uterus was then placed back into the abdominal cavity, and the abdomen wall and skin were sutured. The pregnant mouse was placed on a heating pad until it became conscious, and the embryos were allowed to develop *in utero* until when ready for the experiment.

### Primary culture and post-cultural process

Transfected cortices were dissected and cultured as described previously (Jiang et al., 2023). Briefly, the following day after IUE, GFP-positive regions were dissected from dorsal telencephalon in ice-cold HBSS containing 25 mM HEPES Buffer, trypsinized in 0.05% Trypsin solution for 10 min and dissociated by trituration with fire-polished glass pipettes. The cells were resuspended and plated at a density of 6 × 105 cells/ml in Neurobasal medium containing 2% B27 supplement and 1% penicillin–streptomycin (hyclone), in dishes with glass coverslips coated with poly-ornithine (0.001%) and laminin (5 mg/mL). After 1 ~ 3 days *in vitro* (DIV) at 37°C in 5% CO2, the cells were fixed for 15 min in 4% PFA. And then, immunocytochemistry was carried out for GFP amplification according to IHC procedures.

Cultured neuronal images were traced and analyzed by Neurolucida and Neuroexplorer software (MBF Bioscience). The total length and branch number of each individual process in transfected neurons were analyzed using the program Neuroexplorer.

### Quantification and statistics

The numbers of positive neurons in the images were counted blindly using Fiji/ImageJ (N.I.H., Bethesda, MD). Neurons in upper half of IZ with 1 or 2 processes were recognized as bipolar, and with more than 2 processes were the multipolar. Neurons with Golgi protein GM130 located in the upper quadrant of soma were regarded as up-orientation of Golgi apparatus. All data are presented as mean ± SEM except for additional stated; “N” in each experiment is typically ≥3; Statistics was indicated in the figure legends. *p* values are as indicated in graphs, *p < 0.05, ** *p* < 0.01, *** *p* < 0.001, ns, not significant (*p* ≥ 0.05).

## Results

### Cortical expression of *Slit2* is required for the radial migration of upper-layer projection neurons

To determine the expression pattern of Slit2 in the developing cerebral cortex, we processed two distinct published scRNA-seq datasets from developing cortices (Di Bella et al., 2021; Yim et al., 2024). Upon unsupervised clustering and UMAP visualization, we identified the distribution of different cell type populations according to the expression pattern of cell-type-defining markers (Supplementary Figures S1A, S2A). Interestingly, the precursors (Pax6 positive), intermediate progenitors (Tbr2 positive), and newborn/mature upper-layer projection neurons (Neurod1 and Satb2 positive) forms a consecutive trajectory, aside from the interneurons (Gad2 and Slc32a1 positive) and deep-layer neurons (Fezf2 and Ctip2 positive) (Supplementary Figures S1B, S2B). Such a trajectory potentially indicates the developing process of upper-layer projection neurons. Accordingly, both datasets showed that Slit2 dominantly expressed in the newborn (immature) upper-layer neurons (Supplementary Figures S1B, S2B). We further measured the Slit2 expression in the cortex at multiple developing stages, which showed a relative low-level expression of Slit2 in early developing cortices and the highest expression level in E18.5 cortices (Supplementary Figure S3A).

**Figure 1:**
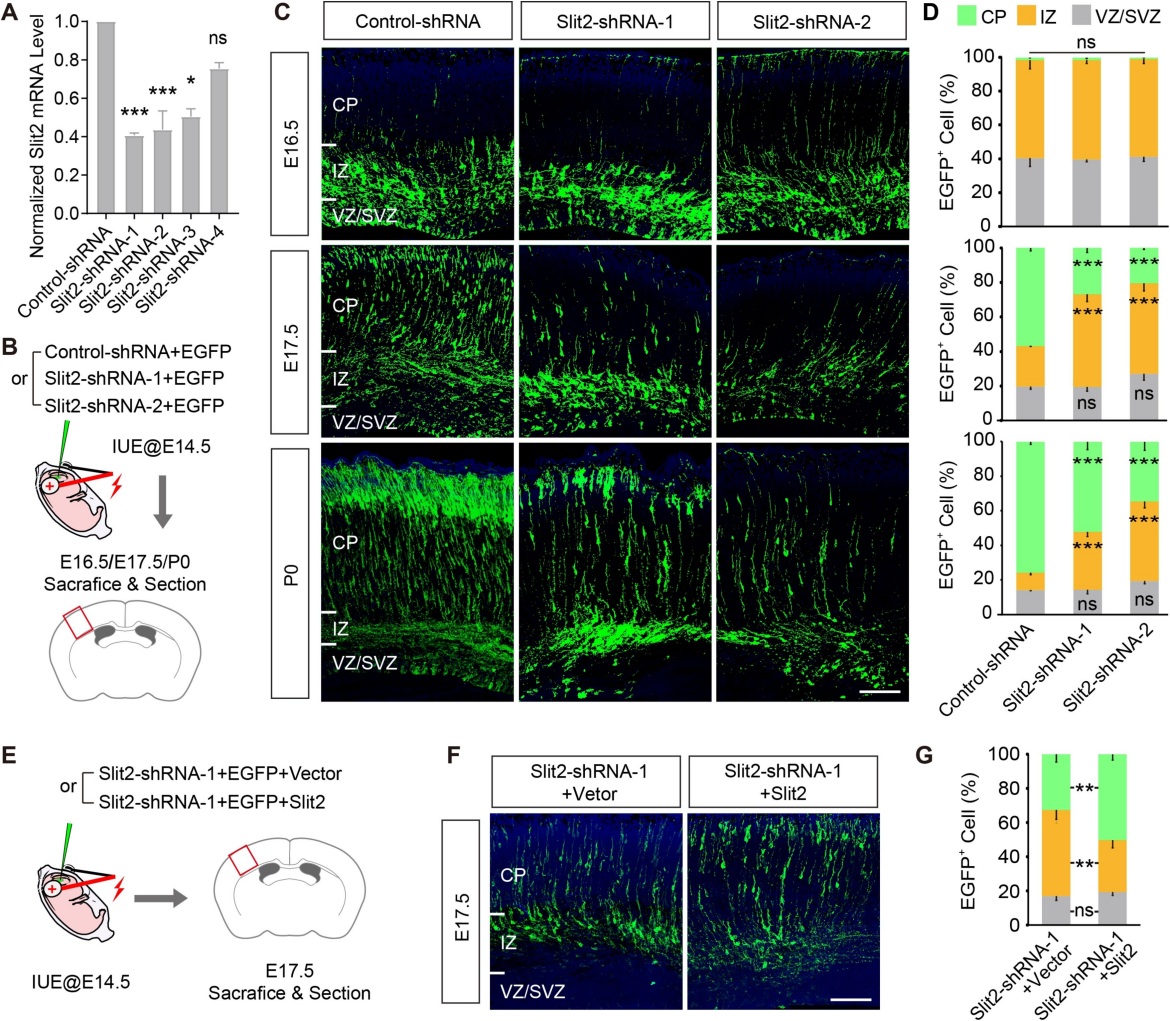
Knocking down *Slit2* results in radial migratory deficiency. **(A)** Inhibitory efficiencies of control-/Slit2-shRNAs, tested in N2a cells. Slit2-shRNA-1, Slit2-shRNA-2 and Slit2-shRNA-3 showed significant efficiency, as compared with control scramble shRNA (Control-shRNA). **(B)** Schematic of the IUE experiments. Embryonic cortices were transfected with Control or Slit2-shRNAs at E14.5, and collected at E16.5, E17.5 and P0, respectively. **(C,D)** Representative images and quantitative data of neuronal radial migration in the neocortex at multiple developmental stages. CP, Cortical Plate; IZ, Intermediate Zone; VZ, Ventricular Zone; SVZ, Subventribular Zone. *N* = 3 mice per group (multiple slices per mice), two-way ANOVA followed by multiple comparisons with two-stage linear step-up procedure of Benjamini, Krieger and Yekutieli correction. Scale bar, 100 μm. **(E)** Schematic of the rescue experiment, co-expressing Slit2 with the Slit2-shRNA. **(F,G)** Representative images and quantitative data of the experiment in **(E)**. *N* = 3 mice per group (multiple slices per mice), two-way ANOVA followed by multiple comparisons with two-stage linear step-up procedure of Benjamini, Krieger and Yekutieli correction. Scale bar, 100 μm. In this and all subsequent figures, data are presented as mean ± SEM, and *N* represents animal number, unless otherwise stated; *p* values are as indicated in graphs, *p < 0.05, ** *p* < 0.01, *** *p* < 0.001, ns, not significant (*p* ≥ 0.05).

To explore the potential role of *Slit2* in regulating radial migration of upper-layer cortical neurons, we knocked down the endogenous *Slit2* expression by *in utero* electroporation of functioning Slit2 shRNAs or its control into the developing neocortex at E14.5 when the upper-layer cortical neurons were generating (Figures 1A,B; Supplementary Figure S3B). The distribution of transfected cells, as labeled by the co-transfected EGFP, in the cortical wall was analyzed at E16.5, E17.5, and P0. At E16.5, most Slit2-shRNA-transfected neurons appeared in IZ, similar to the control group. By E17.5, a large proportion of cells transfected with the control plasmid crossed the IZ and reached the CP. By contrast, far fewer GFP+ cells migrated to the CP in the KD groups (IZ, Control-shRNA 23.4 ± 0.1%, Slit2-shRNA-1 53.6 ± 4.2%, Slit2-shRNA-2 52.6 ± 4.5%; CP, Control-shRNA 56.9 ± 1.7%, Slit2-shRNA1 26.9 ± 2.6%, Slit2-shRNA2 20.4 ± 0.9%) (Figures 1C,D). The difference becomes clearer at P0 when most control cells have entered the CP, while lots of the cells with reduced Slit2 remained accumulated in the IZ (Figures 1C,D). To validate the specificity of Slit2 KD, we performed a rescue experiment by co-expressing the KD shRNA with a gain-of-function plasmid. Overexpression of Slit2 corrected the migration defect of the Slit2-shRNA-transfected cells at E17.5 (Figures 1E–G). Upon observation of the P7 cortex, we found that most of the arrested Slit2-deficient neurons reached their destination eventually. Together, these data suggest that the expression of *Slit2* in the cortical neurons is necessary for their radial migration.

### Knocking down *Slit2* impairs neuronal morphologic re-polarization

**Figure 2:**
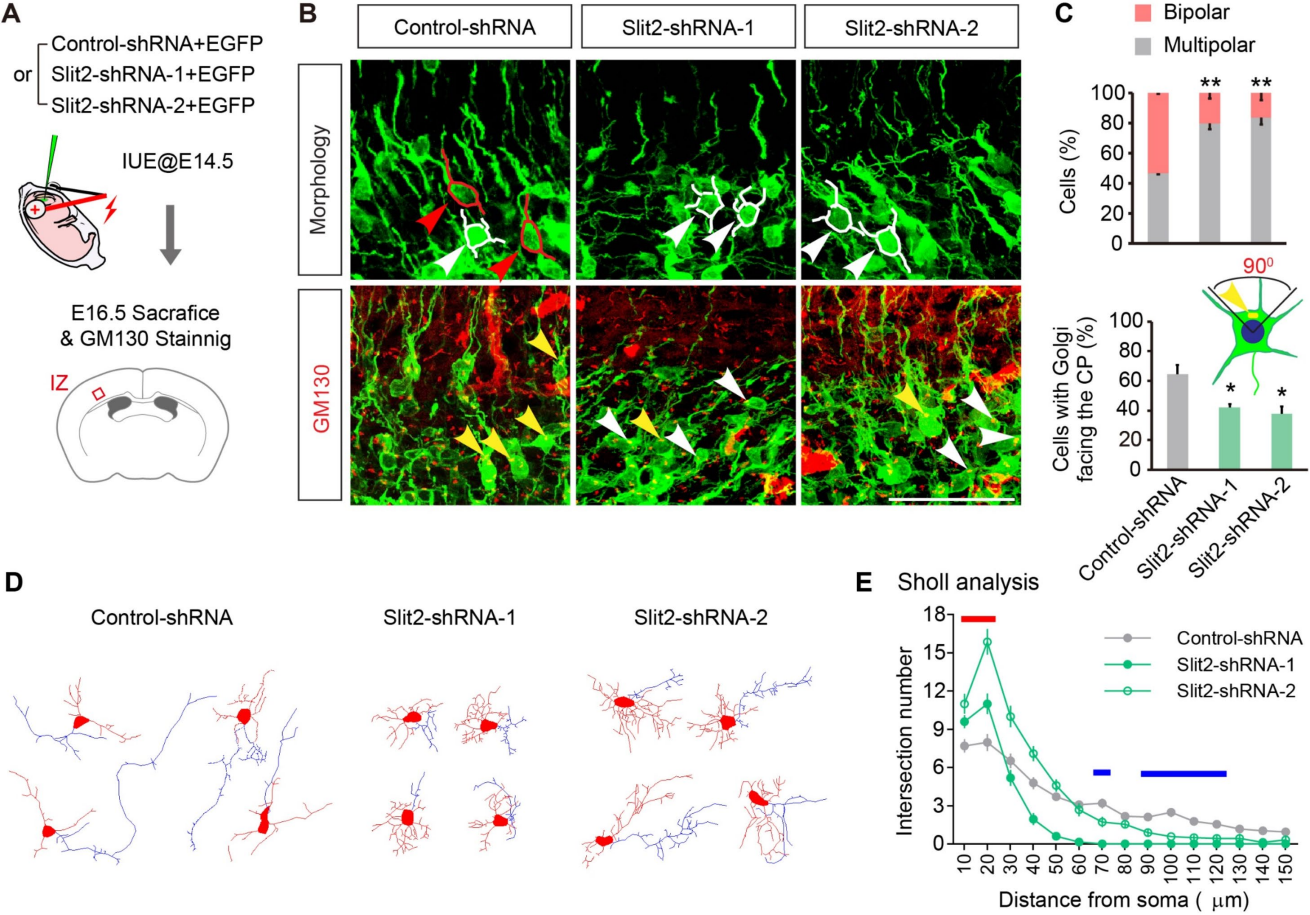
Knocking down *Slit2* leads to deficient morphologic transformation. **(A)** Schematic of the experiment. **(B,C)** Representative images and quantitative data of neuronal morphology and Golgi apparatus orientation in the upper IZ of E16.5 cortices. Red tracings and arrows indicate bipolar neurons, white tracings and arrows indicate multipolar neurons. One-way ANOVA followed by multiple comparisons with two-stage linear step-up procedure of Benjamini, Krieger and Yekutieli correction. Scale bar, 100 μm. **(D,E)** Representative traces **(D)** and morphological quantification data (Sholl analysis, **E**) of cultured neurons transfected with Control or Slit2-shRNAs. *N* = 28 neurons in Control-shRNA group, *N* = 20 neurons in Slit2-shRNA-1 group, *N* = 22 neurons in Slit2-shRNA-2 group. Two-way ANOVA followed by multiple comparisons. The red line indicates distances that both Slit2-shRNA1 and 2 groups showed increased neurites, the blue lines indicate distances that both Slit2-shRNA-1 and -2 groups showed decreased neurites.

Upon inspecting the migratory delay, we noticed that the delayed neurons were mainly detained in the IZ, where the neurons underwent critical morphological re-polarization prior to migrating into the CP. To determine whether the KD of Slit2 disrupts the multipolar-bipolar transition, we examined the neuronal morphology of control and *Slit2-*suppressed neurons in the IZ at E16.5. The migrating neurons in IZ had either multiple short processes or a bipolar shape with a leading (future dendrite) and a trailing (nascent axon) process. KD of *Slit2* substantially increased the number of multipolar neurons while decreasing the ratio of bipolar cells (Figures 2A–C, bipolar ratios: Control-shRNA 53.4 ± 0.8%, Slit2-shRNA-1 20.1 ± 3.8% and Slit2-shRNa-2 16.4 ± 4.1%). We further examined the orientation of the Golgi apparatus by Golgi-specific GM130 immunostaining (Jossin and Cooper, 2011). By the end of the multipolar-bipolar transition, the Golgi apparatus normally moves toward the upper quadrant of cells facing the pial surface. However, KD of *Slit2* reduced the proportion of cells with CP-orienting Golgi apparatus as compared to the control (Figures 2A–C, Control-shRNA: 64.4 ± 6.5%, Slit2-shRNA-1: 41.9 ± 2.3%, Slit2-shRNA-2: 37.7 ± 3.8%). We also conducted the *Slit2* KD experiment in cultured neurons to determine the morphological changes. Consistently, Slit2-deficient neurons showed significant polarization abnormity compared to the control. Sholl analysis confirmed that neurons with Slit2 KD had more short neurites but lacked a dominant axon-like process (Figures 2D,E). These results indicate that *Slit2* knockdown disturbs morphological re-polarization and orientation of cortical neurons.

### *Slit2* expression is not required for neurogenesis and fate determination of cortical projection neurons

**Figure 3:**
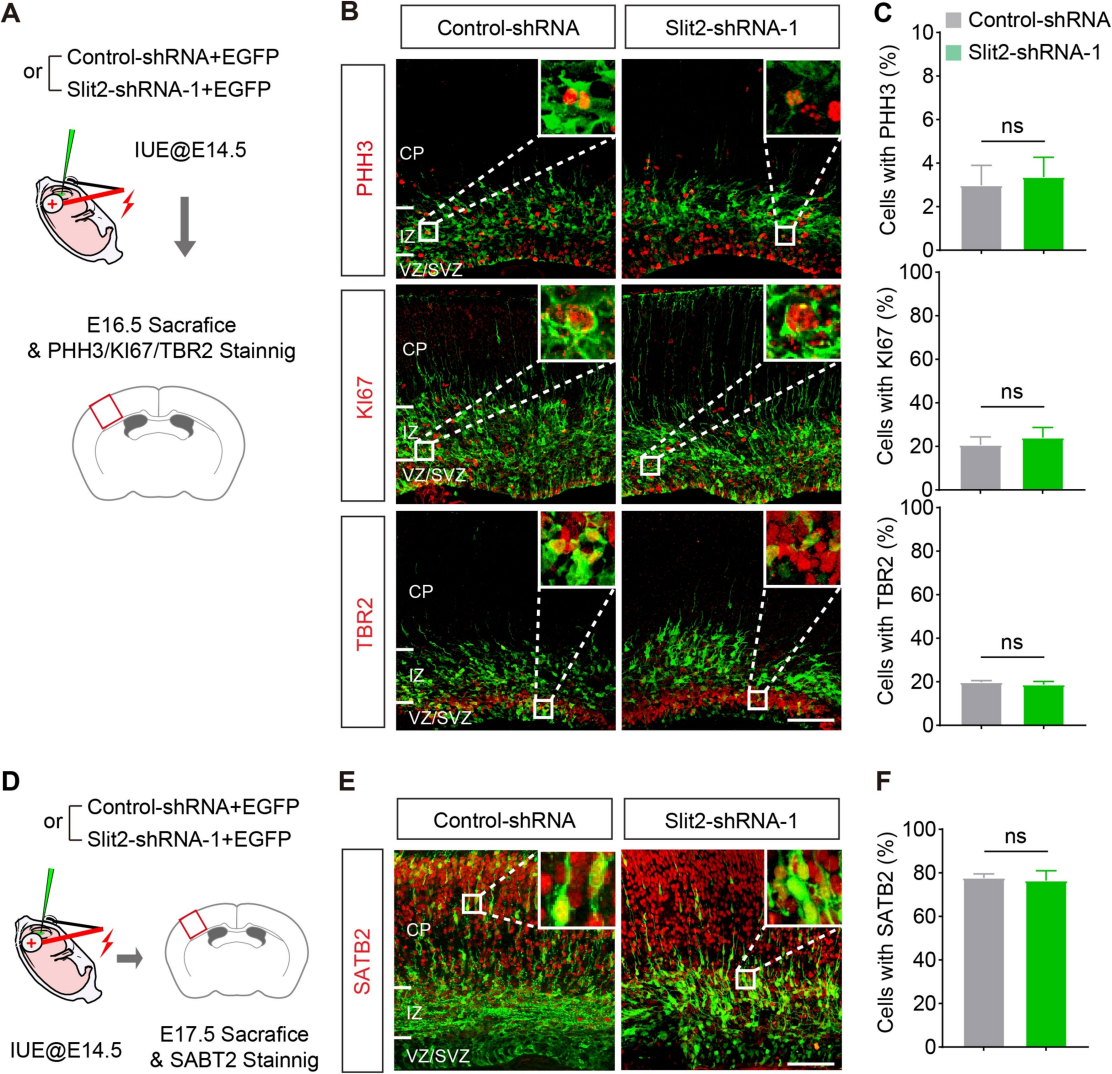
Knocking down *Slit2* does not change neuronal generation and fate. **(A)** Schematic of the experiment. **(B,C)** Representative images and quantitative data of neuronal progenitor (TBR2) and proliferation (PHH3 and KI67) markers in Slit2-deficient neurons. *N* = 4 slices from 2 mice in control-shRNA group, *N* = 5 slices from 2 mice in Slit2-shRNA1 group; Student’s *t*-test. Scale bar, 100 μm. **(D–F)** Same as **(A–C)** but for the postmitotic marker of upper layer-neurons in the neocortex. *N* = 3 slices from 2 mice per group; Student’s *t*-test. Scale bar, 100 μm.

Abnormality in neurogenesis in the VZ/SVZ could lead to the migration delay. To examine this possibility, we stained the neurons with antibodies against phospho-Histone H3 (PHH3, a marker of cell mitosis), KI67 (a marker of cell proliferation) and TBR2 (a marker of intermediate progenitors in the SVZ) in the E16.5 brain slices (Figure 3A). Quantitative analysis showed that *Slit2* inhibition did not change the fraction of mitosis and proliferation cells in the transfected population (PHH3: Control-shRNA 3.0 ± 0.9%, Slit2-shRNA1 3.4 ± 0.9%; KI67: Control-shRNA 20.7 ± 3.7%, Slit2-shRNA1 24.1 ± 4.6%), neither altering the generation of intermediate progenitors (TBR2: Control-shRNA 19.8 ± 0.7%, Slit2-shRNA1 18.7 ± 1.4%) (Figures 3B,C), supporting that the inhibition of Slit2 does not change the cell cycle exit or neurogenesis of cortical upper-layer projection neurons.

To determine the identities of the ectopic cells in IZ after *Slit2* KD, we immuno-stained the brain sections with SATB2, a marker of postmitotic projection neurons in the upper layers of the cerebral cortex (Figures 3D–F). As expected, most of the neurons receiving the control plasmid expressed SATB2. The Slit2-deficient neurons also expressed SATB2 at a comparable ratio as the control, despite that they were mainly located in the IZ (SATB2: Control-shRNA 77.7 ± 1.8%, Slit2-shRNA1 76.4 ± 4.5%). These results indicates that *Slit2* KD does not impair the generation and fate determination of cortical upper-layer projection neurons, which is consistent with a previous observation that knockout of *Slit2* alone has no effect on the cortical neurogenesis (Borrell et al., 2012).

### Cell-autonomous action of Slit2 via ROBO2

**Figure 4:**
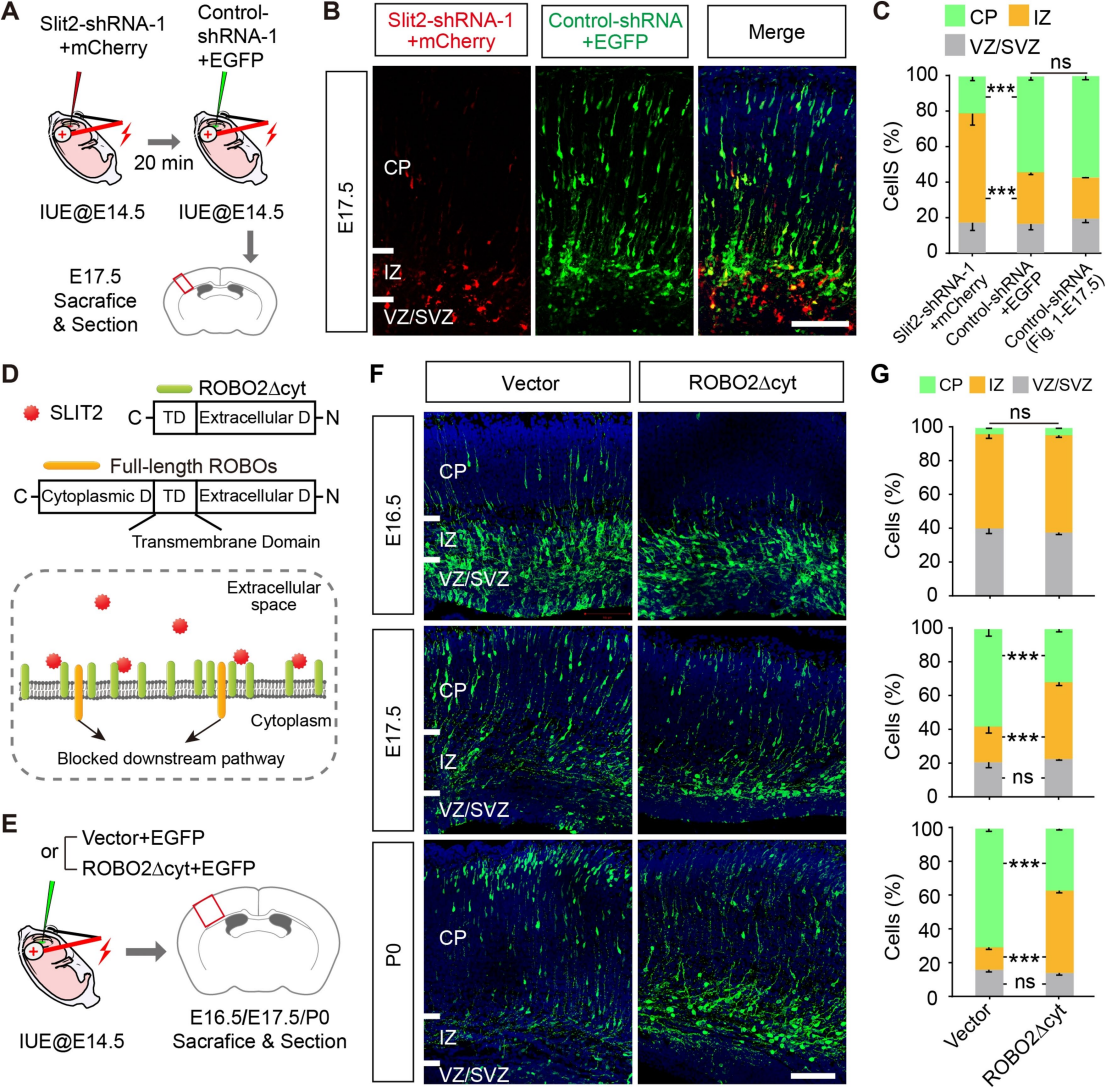
*Slit2* regulates radial migration cell-autonomously via ROBO2. **(A–C)** Schematic of sequential electroporation experiments. **(B,C)** Representative images and quantitative data of two adjacent neuronal populations, transfected with Slit2-shRNA-1 + mCherry or Control-shRNA+EGFP. Control-shRNA data from Figure 2. E17.5 were shown as a standard control. Two-way ANOVA followed by multiple comparisons with two-stage linear step-up procedure of Benjamini, Krieger and Yekutieli correction. Scale bar, 100 μm. **(D)** Schematic of ROBO2∆cyt and the working model for acting as a competitive extracellular inhibitor of SLIT-ROBO signaling. **(E–G)** Experimental procedures, representative images and quantitative data of the radial migration profiles of ROBO2∆cyt-expressing neurons. *N* = 4 per group in E16.5, *N* = 3–4 per group in E17.5, *N* = 2–3 per group in P0, two-way ANOVA followed by multiple comparisons with two-stage linear step-up procedure of Benjamini, Krieger and Yekutieli correction. Scale bar, 100 μm.

The above results suggest that the expression of Slit2 in the cortical neurons is required for radial migration. Given that SLIT2 is a secretory protein and functions by activating its canonical receptors ROBOs (Dickson and Gilestro, 2006), we investigated whether Slit2 deficiency affects the migration of neighboring neurons. Thus, we electroporated Slit2-shRNA-1 + mCherry into upper layer projection neurons at E14.5 and then labeled the adjacent neurons with Control-shRNA+EGFP 20 min later (Figure 4A). After sequential electroporation, the brains were harvested at E17.5. Interestingly, many neighboring neurons showed a normal migration pattern, in contrast to the neurons with *Slit2* KD that were stalled at IZ, suggesting the effect of *Slit2* KD was limited to the cells that received the shRNA (Figures 4B,C).

The cell-autonomous effect could be mediated by intracellular SLIT2 or via canonical Robo-dependent secretory pathway by autocrine. To discriminate it, we constructed a truncated ROBO2 (ROBO2∆cyt) that lacks the cytoplasmic part of ROBO2 [acting as a competitive extracellular inhibitor of SLIT-ROBO signaling as previous studies reported (Wu et al., 1999; Whitford et al., 2002)], to test the contribution of extracellular SLIT signal (Figure 4D). We then introduced ROBO2∆cyt into the cortex at E14.5 and examined the positioning of cortical neurons at E16.5, E17.5, and P0 (Figure 4E). ROBO2∆cyt-transfected neurons showed migratory delay (Figures 4F,G), similar as the effect of Slit2-shRNAs, suggesting an autocrine SLIT2 signaling in the process. In addition, among the Slits and Robos, only ROBO2 showed comparable expression pattern as Slit2, as shown by the scRNA-seq data (Supplementary Figures S1C, S2C). These results indicate that the SLIT2-ROBO2 signaling pathway may contribute to regulate radial migration, and SLIT2 likely acts as a secretory molecule via autocrine.

## Discussion

In vertebrates, SLIT2 is a chemorepellent that guides the development of axons (Dickson and Gilestro, 2006). A*xon guidance and neuronal migration, two* fundamental processes in brain development, frequently share common mechanisms of action. For example, Sema3A regulates both axon guidance and radial migration of cortical neurons via Rho-family GTPases (Chen et al., 2008). In the present study, we found that suppression of Slit2 *in vivo* leads to a defect in radial migration of the cortical neurons, and the defect arises from disrupted polarization associated with Golgi orientation (Figure 1). This study complements previous findings on the role of Slit2 in cellular migration (Hu, 1999; Xu et al., 2004; Chen et al., 2008; Xu et al., 2010; Bielle et al., 2011; Feng et al., 2016; Zeng et al., 2018). Furthermore, we found that extracellular ligand-dependent activation of ROBOs is required for radial migration (Figure 4), suggesting that SLIT2 secretion and interaction with its membrane receptor are the fundamental processes involved in the regulation of radial migration.

Electroporation only transfected a small portion of cortical neurons. Thus, KD of Slit2 may not have caused a significant reduction of this secretory molecule in the extracellular space of the cortical plate. However, KD led to a migration delay of the specific neurons that received the shRNA (Figure 2). This supports that Slit2 regulates radial migration locally but not remotely. Slit2 is a ligand of heparan sulfate proteoglycan glypican-1 and interacts with the extracellular matrix (ECM) (Liang et al., 1999), which may limit its diffusion in the brain. In zebrafish, tectum-derived Slit1 presents laminar positional cues to ingrowing retinal axons by binding to a type IV Collagen (Xiao et al., 2011). In the mammalian nervous system, Slit2 binds directly to dystroglycan, and the proper Slit2 localization within the basement membrane is a key determinant of axon guidance cue distribution (Wright et al., 2012). In addition, autocrine and juxta paracrine regulation contributes to motor axon fasciculation by Slit-Robo signaling via the ECM (Jaworski and Tessier-Lavigne, 2012). The cell-autonomous action of Slit2 has also been reported in dendrite self-avoidance of cerebellar Purkinje cells (Gibson et al., 2014). We speculate that Slit2 may regulate radial migration of cortical neurons cell-autonomously by binding to ECM.

Our results also illustrated the potential downstream mechanism for the regulation of Slit2 in radial migration. Both *in vivo* and *in vitro* experiments demonstrated that Slit2 knockdown impairs neuronal morphogenesis, rather than neurogenesis. Specifically, Slit2 may modulate radial migration via controlling the Golgi apparatus orientation and neuronal re-polarization. Interestingly, the small GTPases (e.g., Cdc42, downstream molecules of SLIT-ROBO) have been reported involved in the orientation and polarization of multipolar neurons in the developing neocortex (Jossin and Cooper, 2011). Given the involvement of Slit-Robo signaling and the expression patterns of Robos (Supplementary Figures S1, S2), we assume that Slit2 modulates radial migration by regulating neuronal orientation and re-polarization via canonical Slit2-ROBO2-small GTPases signaling pathway. Nevertheless, the mechanical proposal lacks casual evidence currently and needs to be verified by further study.

Neuronal migration is critical for assembling neuronal circuits in the cortex, and abnormal neuronal migration is the underlying pathophysiology for neurological and psychiatric disorders. Developmental dyslexia is a specific childhood learning disorder. SLIT2 was proposed as a candidate for developmental dyslexia (Poelmans et al., 2011). In addition, ROBO1 is also linked to the developmental dyslexia (Hannula-Jouppi et al., 2005). Our result suggests that an inherited defect in SLIT2 may impair the radial migration and proper formation of the neocortex, which may contribute to developmental dyslexia. Thus, this study improves our understanding of SLIT2’s role in the developing brain and may shed light on the underlying mechanisms of SLIT2-related neuronal disorders.

## Data Availability

The original contributions presented in the study are included in the article/Supplementary material, further inquiries can be directed to the corresponding authors.

## Footnotes

^1^ http://www.sigmaaldrich.com
